# Supplementary material for: Learning curve and surgical outcome of robotic assisted colorectal surgery with ERAS program
Source: Sci Rep. 2022 Nov 29;12:20566. doi: 10.1038/s41598-022-24665-w (PMC9709162; doi:10.1038/s41598-022-24665-w)
Supplement: Supplementary file 1 — Supplementary Information 1. [file 41598_2022_24665_MOESM1_ESM.zip › LSC Raw data-Ñ[▒K/135/H_ñw╝╨░O▒Kñσ.pdf]

正式摘要(電子簽章：0000002255110558)<br>W83 / W83 ☐ 英文標題(注意:無英文姓名-請補病人基本檔!)	☐【讀取】憑證	

臺中榮民總醫院
出院病歷摘要

轉入號： 轉出號：

[redacted]

(8)流水編號	
(9)入院日期	110/08/25 (2021) 直腸外科 W83 - 102
(10)轉科日期	
(11)出院日期	110/09/11 (2021) 住院天數計 17 日

診斷

- 入院：1. Rectal cancer with liver metastasis
- 出院：1. Adenocarcinoma of rectum, moderately differentiatedwith liver metastases(S6, deep S5)
  - s/p laparoscopic LAR with anastomosis+ laparoscopic S5, S6 subsegment hepatectomies on 2021/08/27

主訴

- Rectal tumor

病史

- This is a 63 year-old man, denied any systemic diease before.
  This time, he had accidently found rectal tumor. According to the patient, he receive Stool OB examination, and reveal positive. Due to those, he receive colonscope and rectal tumor was noted. Abdominal CT was done and reveal liver nodule.
  Recently, he had poor appetite, bowel habit change, body weight loss for 5-6kg within recent 3 months. Due to those, he went to our CRS OPD for help. Under impression of rectal cancer with liver metastasis, he was admitted for further surgical intervention.

體檢發現

-
  - General appearance :
    - easy looking , conscious clear , without cardiopulmonary distress
    - Vital signs自動帶入date 體溫(BT) 脈搏(P) 呼吸(R) 血壓(BP) 身高(BH) 體重(BW) BMI
      20210825 35.9℃ 67次/分 18次/分 119/77mmHg 164cm 61.4kg 22.8
    - Vital signs: B.P. 119/77 mmHg , P.R. 67 /min , R.R. 18 /min , B.T. 35.9 ℃
  - HEENT :
    - Eyes: no anemic conjunctiva, no icteric sclera, normal EOM
    - ENT: no otorrhea, no rhinorrhea, no erythematous mucosa, no injected tonsil, no deformity
  - Neck: supple, freely movable, trachea midline,no deformity, no engorged jugular vein , no palpable thyroid or lymph node enlargement
  - Chest:
    - Inspection: Grossly normal, symmetric and fully expansion, no accessory muscle used, no operation scar, no spider angiomata, no gynecomastia
    - Palpation: no palpable mass or nodule, no subcutaneous emphysema, no tenderness
    - Ausculation: clear breathing sound
  - Heart:
    - Percussion: normal heart size
    - Ausculation: regular heart beat, normal S1, S2, no gallop, no murmur
  - Abdomen:
    - Inspection: in
      - flat contour, symetrically, no scar, no stria, no dilated veins, no rashes
    - Ausculation: normoactive bowel sound, no abdominal bruits

-
    - palpation: soft, no muscle guarding or rigidity, no local tenderness, no rebounding pain, no palpable mass, no palpable pulsation
    - Percussion: no shifting dulllness, no hepatomegaly, no splenomagaly
-

Peripheral pulsation :	CA	BA	RA	FA	PA	PTA	DPA
Right side :	++	++	++	++	++	++	++/+++
Left side :	++	++	++	++	++	++	++/+++

- Genito-anus: no tenderness or palpable mass lesion or rectal shelf, normal external genitalia, normal anal tone, brown stool

CRS CONDITION

-
    - Lung

手術日期及方法

- 手術日期及方法20210827 1. Laparoscopic low anterior resection with loop ileostomy
  2. Intraoperative colonoscopy : (-)
  3. High ligation (+) : At IMA root. IMV and left colic artery were also ligated.
  4. EndoGIA (60-4.1 ; Green) X4 + EEA (29 mm) were used
  5. Proximal cut end: 15 cm, distal cut end: 2 cm
  6. Loop ileostomy was created and fixed over RLQ of abdomen (About 20 cm prior to cecum).
  7. One 19 Fr. hemovac tube was placed from LLQ of abdomen and inserted into the pelvic cavity
  8. The colon was taken down from the splenic flexure site
  20210827 1.LIVER PARTIAL HEPATECTOMY2.Biliary operative sonogram3.Laparoscopy4.
- 手術結果及發現 :
    - 1. A 3 x 3 cm, ulcerative tumor at lower rectum. (DRE : Finger tip) 2. No other gross tumor nor polyps was found 3. There were obvious visible liver metastasis, S5, S6 under laparoscope and intraoperative sonography; no visible peritoneal seeding. 4. No obvious ascites was noted over the pelvic area. 5. Bilateral doughnuts : intact ; Air leak test: Done and no air leak was found

住院治療經過

- After admission, preoperative prepare was done, he underwent laparoscopic LAR with anastomosis+ laparoscopic S5, S6 subsegment hepatectomies smoothly on 2021/08/27. After operation, he had regained conscious and tolerated with pain control. He had tried oral diet smoothly. Loose stool formation was noted from ileostoma. Lab data showed no abnormality. Under his relative stable condition, he was discharged on 09/11.

合併症

- Nil

一般檢查

- 累積報告 :

DATE	TIME	ALB	TP	BIL.T	BIL.D	ALKP	AST	ALT	LDH	AMY	LIPASE	NH3	Ferritin	UA
1100827	2203	3.0		0.88				260						
1100830	0920	3.0		0.62	0.24	61	33	92						
1100902	1500	2.7		0.34	0.15		26	23						
1100906	0913	2.9		0.25				14						
1100909	0910	2.8		0.31	0.14		18	11						

DATE	TIME	WBC	RBC	HGB	HCT	MCV	PLT	BLST	NEUT	BAND	SEG	LYM	MON	EOS	BAS
1100827	2202	20070		11.0	33.1		307				93.0	4.0	3.0	0.0	0.0
1100830	0924	14450	3.87	11.3	34.4		234		88.9			5.7	5.1	0.1	0.2
1100902	1459	9320	3.95	11.7	33.8	85.6	347		73.2			12.4	10.1	3.9	0.4
1100906	0947	6270		11.8	35.5		428		66.7			18.3	10.0	4.0	1.0
1100909	0909	6440	3.98	11.6	35.0	87.9	448		71.0			16.9	7.1	4.2	0.8

DATE	TIME	NA	K	CL	BUN	CREAT	eGFR	CA	Mg	P	CRP	ESR	PCT	D-Dimer	LACTATE
1100827	2203	143	3.4	106	9	0.75	111.8	7.2							
1100830	0920	136	3.3	102	10	0.73	115.34	7.3							
1100902	1500	136	3.8		9	0.61	141.9	7.4							
1100906	0913	138	4.1		8	0.71	119.1								
1100909	0910	136	3.9		5	0.66	129.57	7.4							

- 放射線報告 :**檢驗項目 :ABDOMEN(KUB),STADING** 報告日期 **:1100908**
  主　訴 :
  Result:N
  Finding:
  > Some gaseous bowel loop dilatation in the abdomen, R/I ileus
  > Bilateral psoas shadows and kidney contours are partially obscured by bowel gas

- > Degenerative change of T-L spine with osteophyte formations
  > Surgical clips retention over RUQ of abdomen
  Impression:
  As above description.

特殊檢查

病理報告

- 活體組織切片病理報告 手術日期：**110/08/27** 病理號：**S1026843**

診斷醫師:吳致瑩 報告日期:110/09/07
Pathologic diagnosis:
1. Rectum, laparoscopic low anterior resection --- a. Adenocarcinoma, moderately differentiated. Tumor invaded to mesocolic soft tissue. The bilateral cut ends and mesocolic lymph nodes (0/16) are free of tumor b. Tubular adenoma.
2. IMA root, dissection --- Unremarkable fibroadipose tissue and lymph nodes (0/3).
3. Rectum, cut end, distal, resection --- Unremarkable.
4. Liver, S5, biopsy --- Metastatic adenocarcinoma, favored colonrectal origin.
5. Liver, S6, biopsy --- Metastatic adenocarcinoma, favored colonrectal origin. The section margin is 1 mm to tumor.

Ancillary study for diagnosis:
1. IHC stains for SATB2 (+) done on section C.
2. IHC stains for PMS2 and MSH6 are done on section C. Both markers are preserved.

Prognostic and predictive factor:
1. Tumor size: 4x3.5 cm.
2. Depth of invasion: Pericolorectal tissue.
3. Histologic grade: G2: Moderately differentiated.
4. Angiolymphatic invasion: Not identified.
5. Perineural invasion: Present.
6. Tumor deposits (discontinuous extramural extension): Present, specify number of deposits: 1.
7. Macroscopic tumor perforation: Not identified.
8. Surgical Margins: a) Proximal Margin: Uninvolved by invasive carcinoma, distance of tumor from margin: 15 cm. b) Distal Margin: Uninvolved by invasive carcinoma, distance of tumor from margin: 1.8 cm. c) Radial or Mesenteric Margin: Involved by invasive carcinoma (tumor present 0-1 mm from margin).
9. Lymph node status: Uninvolved (0/19).
10. Extranodal involvement: Not identified.
11. Tumor regression grading S/P CCRT: No known presurgical therapy.
12. Type of polyp in which invasive carcinoma arose: Not identified.
13. Additional pathologic findings: Adenoma.
14. Pathological TNM Stage: pT3N1cM1a(According to the eighth edition, American Joint Committee on Cancer Staging Guidelines for Tumors).
15. TNM descriptors: Not applicable.

Gross description:
The specimen consists of 1) a segment of rectum, 21.0 cm in length and 7.0 cm in proximal cut end circumference, 5.0 cm in distal circumference, and perirectal soft tissue. A gray tan firm annular elevated and centrally ulcerative tumor, 4.0x3.5 cm, is noted, 1.8 cm to the proximal cut end. On cross section, the tumor invades to the perirectal soft tissue with 3 mm to the radial margin, and serosal retraction is also noted. A polyp, 0.4x0.3x0.3 cm, 8 cm to the proximal cut end, is seen. The both cut ends are free of tumor grossly. 2) IMA root, 3.8x3.2x1.0 a piece of tan yellow soft tissue, cm. 3) distal cut end, a piece of tan mucosa-covered soft tissue, 2.2x1.5x0.5 cm. 4) liver S5, 7.0x7.0x2.4 cm, the specimen has been cut when received, a yellow tan nodule, 0.8x0.8x0.5 cm, is noted. 4) liver S6, 6.2x5.4x1.3 cm. A yellow tan nodule, 2.8x2.0x1.4 cm, is noted when serial cut. Representative part for section: A) distal cut end B) proximal cut end C-D) tumor E) polyp F) non tumor rectum G) specimen 2 H) specimen 3 J) specimen 4 K) specimen 5 L-N) lymph nodes.

- Note:
  1. The interpretation of MMR panel and recommended studies are as below: (1) No loss of nuclear expression of MMR proteins: low probability of microsatellite instability-high (MSI-H) . (2) Loss of nuclear expression of MLH1 and PMS2: testing for methylation of the MLH1 promoter and/or mutation of BRAF is indicated (the presence of a BRAF V600E mutation and/or MLH1 methylation suggests that the tumor is sporadic and germline evaluation is probably not indicated; absence of both MLH1 methylation and of BRAF V600E mutation suggests the possibility of Lynch syndrome, and sequencing and/or large deletion/duplication testing of germline MLH1 may be indicated). (3) Loss of nuclear expression of MSH2 and MSH6: high probability of Lynch syndrome (sequencing and/or large deletion/duplication testing of germline MSH2 may be indicated, and, if negative, sequencing and/or large deletion/duplication testing of germline MSH6 may be indicated). (4) Loss of nuclear expression of MSH6 only: high probability of Lynch syndrome (sequencing and/or large deletion/duplication testing of germline MSH6 may be indicated). (5) Loss of nuclear expression of PMS2 only: high probability of Lynch syndrome (sequencing and/or large deletion/duplication testing of germline PMS2 may be indicated). (6) Please note: There are exceptions to the above IHC interpretations. These results should not be considered in isolation, and clinical correlation with genetic counseling is recommended to assess the need for germline testing.
  #T-59300_2 #M-81403_2 2 1105 S0000F
  #T-62000_2 #M-81406_2 2 1104 000000
  #T-C4560_2 #M-09183_2 0 1105 000000

其它

出院時情況

- 3.改門診治療

出院指示

藥名	劑量	劑量單位	用法	頻次	數量	數量單位	現況	天數	單位
Otilonium tab 40mg	1	TAB	PO	TID	15	TAB	TH	5	天
Loperamide cap 2mg	1	CAP	PO	BIDPRN	10	CAP	TH	5	天
Ultracet tab	1	TAB	PO	BIDPRN	10	TAB	TH	5	天

出院計劃

- 門診日期
  - date on 2021/09/15

主治醫師_2062F 蔣鋒帆_ / 住院醫師_2336L 洪浚嚴_

正式摘要(電子簽章：000000225511058)

W83 / W83

doctorRank:B
